# The Role of Neurod Genes in Brain Development, Function, and Disease

**DOI:** 10.3389/fnmol.2021.662774

**Published:** 2021-06-09

**Authors:** Svetlana Tutukova, Victor Tarabykin, Luis R. Hernandez-Miranda

**Affiliations:** ^1^Institute of Neuroscience, Lobachevsky University of Nizhny Novgorod, Nizhny Novgorod, Russia; ^2^Charité Universitätsmedizin Berlin, corporate member of Freie Universität Berlin and Humboldt-Universität zu Berlin, Institute for Cell- and Neurobiology, Berlin, Germany

**Keywords:** bHLH factor, neurod family, brain development, neurological diseases, transcription factors

## Abstract

Transcriptional regulation is essential for the correct functioning of cells during development and in postnatal life. The basic Helix-loop-Helix (bHLH) superfamily of transcription factors is well conserved throughout evolution and plays critical roles in tissue development and tissue maintenance. A subgroup of this family, called neural lineage bHLH factors, is critical in the development and function of the central nervous system. In this review, we will focus on the function of one subgroup of neural lineage bHLH factors, the Neurod family. The Neurod family has four members: Neurod1, Neurod2, Neurod4, and Neurod6. Available evidence shows that these four factors are key during the development of the cerebral cortex but also in other regions of the central nervous system, such as the cerebellum, the brainstem, and the spinal cord. We will also discuss recent reports that link the dysfunction of these transcription factors to neurological disorders in humans.

## Introduction

The interest to understand the molecular mechanisms that generate our central nervous system has never been greater, as the intensive work of clinicians, neurologists, and developmental biologists demonstrate that several naturally occurring neurological disorders originate from deficits impairing brain development in humans (Ross and Walsh, 2001; Subramanian et al., 2019). This can be particularly seen in disorders affecting the development of the cerebral cortex, which are frequently associated with seizures both in childhood and in adult life (Subramanian et al., 2019). Furthermore, cognitive disorders ranging from mild to severe intellectual disability and autism are also concomitant features of cortical neurodevelopmental disorders (Guerrini and Dobyns, 2014). The advent of novel and powerful human genetics is greatly contributing to the identification of rare and common disease-causing variants disrupting the normal development of the nervous system (Ku et al., 2010; McCarthy and MacArthur, 2017; Niemi et al., 2018; Momozawa and Mizukami, 2021). Many of these underlie the elementary mechanisms acting on neurogenesis, neuronal differentiation, fate acquisition, dendritogenesis, axonal navigation, and synapse formation (Cardoso et al., 2019; Wang et al., 2019; Parenti et al., 2020).

Development of the central nervous system in humans, as in many other species, is an elaborated process that begins during an early fetal stage, for instance in the third gestational week in humans or by embryonic day 11 in mice (Bayer and Altman, 2007). It initiates with the formation of the neural tube and the differentiation and specification of neural progenitor cells that, subsequently, lead to the genesis of differentiated neurons in a process called neurogenesis that culminates in the early postnatal life in humans, but can span throughout the adult life in other species, such as rodents (Altman and Das, 1965; Johnson, 2001; Bayer and Altman, 2007; Stiles and Jernigan, 2010; Silbereis et al., 2016; Sorrells et al., 2018; Buffalo et al., 2019; Petrik and Encinas, 2019). The specification of neural progenitor cells and their activation to self-renew and/or to differentiate in more committed progenitors and neurons is mediated by extrinsic and intrinsic molecular mechanisms (Götz and Sommer, 2005; Urbán and Guillemot, 2014; Götz et al., 2016; Oproescu et al., 2021). The intrinsic mechanisms that direct neural progenitor cell progression and differentiation rely on the coordinated function of multiple transcription factors that determine their identity and, simultaneously, the suppression of their progenitor cell programs (Schuurmans et al., 2004; Britz et al., 2006; Hevner et al., 2006; Davidson, 2010; Hodge and Hevner, 2011; Busskamp et al., 2014; Ware et al., 2016; Mall et al., 2017; Lee et al., 2019).

In the developing nervous system, proneural basic Helix-loop-Helix (bHLH) transcription factors are master regulators of cell proliferation and key in neuronal differentiation and specification (Dokucu et al., 1996; Sommer et al., 1996; Bertrand et al., 2002). Among these factors, the Neurod family stands as a critical regulator of neuronal progenitor cell differentiation and neuronal specification in the cerebral cortex, as well as in other regions of the nervous system such as the cerebellum, the brainstem, and the spinal cord. The Neurod family is composed of four members, Neurod1, Neurod2, Neurod4, and Neurod6.

In this review, we will discuss on the function of bHLH factors in neuronal development and particularly focus on the Neurod family in the development of cerebral cortex (including neuronal fate specification, dendritogenesis, and axonal navigation), as well as on the function of these factors in the development of other areas of the central nervous system. In addition, we will discuss *Neurod* disease-causing variants found in patients presenting with neurological disorders, such as Alzheimer’s disease.

## bHLH Transcription Factors and The Neurod Family

The bHLH superfamily of transcription factors contains numerous genes crucial for the regulation of gene expression in most eukaryotic organisms. These factors are classified according to the similarities in their protein structure and the characteristic presence of a basic domain that directly binds to chromatin as well as a Helix-loop-Helix (HLH) domain that comprises of a non-conserved loop region connecting to alpha-helices (Chien et al., 1996; Bertrand et al., 2002). The bHLH domain was first identified by Murre and colleagues using early, but sophisticated, oligonucleotide screening procedures on lgt11 expression clones (Murre et al., 1989). The protein sequence characteristic of the bHLH domain consists of about 60–100 amino acids. bHLH factors are known to heterodimerize with other bHLH factors, using their non-conserved loop region, to form a functional DNA binding unit. Upon forming heterodimers, bHLH transcription factors are capable to bind to E-box motifs on chromatin, which display the consensus sequence CANNTG (Longo et al., 2008). The central “NN” and flanking nucleotides are believed to confer the DNA-binding specificity shown by bHLH proteins (Ellenberger et al., 1994; Bertrand et al., 2002).

The bHLH superfamily of transcription factors is well conserved throughout evolution and was first identified in animals, although recent investigations have started to reveal their presence and function in other organisms that range from yeast to plants (Murre et al., 1994; Zhang T. et al., 2018). Phylogenetic analysis of the bHLH superfamily using seven different species (human, mouse, rat, worm, fly, yeast, and plant Arabidopsis) has revealed over 600 members belonging to this family (Stevens et al., 2008). Unsurprisingly, the number of bHLH genes increases with the complexity of the organism, for instance, the smaller number of bHLH genes, 38, is found in *Caenorhabditis elegans*, around 58 in *Drosophila melanogaster*, 117 in the *Mus musculus*, and approximately 130 in *Homo sapiens* (Ledent et al., 2002; Skinner et al., 2010).

In 1989, Murrey and others first classified bHLH transcription factors according to their expression pattern into two classes: a class A (with ubiquitous expression) and a class B (with tissue-specific expression; Murre et al., 1989). This classification has been further expanded using large–scale phylogenetic analyses comparing the bHLH domains (Sun and Baltimore, 1991; Atchley and Fitch, 1997; Meredith and Johnson, 2000; Dennis et al., 2019). A more recent phylogenetic classification done by Skinner and colleagues has related bHLH factors into five distinct clades, in which clade A contains neural lineage genes such as *Neurod*, *Neurog1*, *Ascl1*, and *Atoh1*; or clade C that contains muscle-specific genes such as *Myod1* or *Myf5* (Skinner et al., 2010).

Neural lineage bHLH transcription factors participate in the regulation of cell survival, differentiation, migration, and fate specification during neural development and in postnatal life. These factors oftentimes have overlapping functions but can be further subdivided into: (i) proneural or determination factors (usually expressed in progenitor cells) and (ii) differentiation factors [predominantly expressed in postmitotic neurons (Bertrand et al., 2002)].

(i) *Proneural factors*. They represent a small subset of the neural lineage bHLH factors which are preferentially expressed in multipotent precursor cells. They control and direct progenitor cell decisions as well as the cellular fate choices to undergo glial or neuronal differentiation. An interesting trait of these transcription factors is their pioneering function to remodel chromatin and their capacity to reprogram non-neuronal differentiated cells into neurons (Wapinski et al., 2013, 2017; Pataskar et al., 2016; Guillemot and Hassan, 2017). Members of this group include the genes *Neurod1*, *Neussrod4*, *Ascl1*, *Neurog1*, and *Neurog2*.

(ii) *Differentiation factors*. These genes encompass most of the neural lineage bHLH transcription factors and are predominantly expressed by differentiated neurons, in which they regulate fate specification and neuronal identity maintenance. Members of this group include *Neurod1*, *Neurod2*, *Neurod6*, *Bhlhe22*.

**Figure 1:**
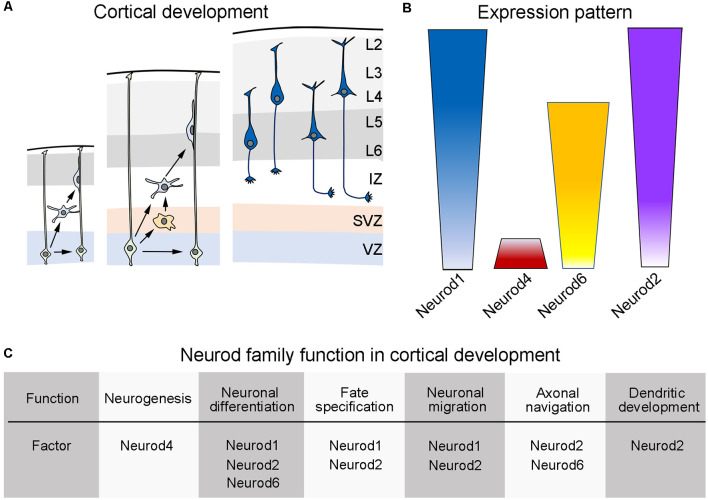
Developmental expression of the Neurod family during cortical development of mice. **(A)** Schematic representation of the cortical development in mice. (Left) During early cortical development, neural progenitor cells (light green, also called radial glial cells) located in the dorsal ventricular zone (VZ) make early decisions as to self-renew or differentiate into early born neurons (light blue). (Middle) As cortical development progress, neuronal progenitor cells increase their choices and can then self-renew or differentiate into more committed progenitors (light orange) that populate an emerging subventricular zone (SVZ) or into differentiated neurons (light blue). Upon differentiation, neurons radially migrate throughout an intermediate zone (IZ) in order to populate the developing cortical plate (gray areas), using radial glial fibers as a scaffold. (Right) According to the time of birth, cortical pyramidal neurons (dark blue) settle into their appropriate cortical layer and start the elaboration of dendritic trees and the elongation or their axonal processes (see text). **(B)** Schematic display of the expression pattern exhibited by the different members of the Neurod family during cortical development (see text). **(C)** Table summarizing the known functions of the different members of the Neurod family during cortical development (see text). Panel **(A)** is inspired from Guo et al. (2015).

The Neurod family contains four closely related proteins: Neurod1, Neurod2, Neurod4, and Neurod6. The expression pattern of these genes is highly overlapping but not identical in the developing cerebral cortex (Figure 1). Expression of these genes is abundantly present in the neuroepithelium of the dorsal telencephalon in early development and is sustained in the adult neocortex, hippocampus, and cerebellum (Schwab et al., 1998). The expression of *Neurod1* can be first detected around embryonic day 12 in the dorsal ventricular zone (VZ) of mice (Bormuth et al., 2013). In the developing cerebral cortex, *Neurod1* is also expressed by mitotic and early-postmitotic neuronal cells that reside in the subventricular zone (SVZ), which contains transit-amplifying progenitors that contribute to the generation of most of the excitatory neurons that form the mature cortex. In the postnatal life, *Neurod1* expression is retained in the cerebral cortex, particularly in most excitatory pyramidal neurons that form the upper-most layers of the cortex (Lee et al., 1995; Bormuth et al., 2013; D’Amico et al., 2013). *Neurod4* expression has been reported to be confined to the ventricular zone of the dorsal telencephalon during development and can be detected around embryonic day 12/13 in mice (Mattar et al., 2008). The other two members of the Neurod family, Neurod2 and Neurod6, display a highly overlapping expression pattern that appears in the mouse cerebral cortex around embryonic day 12 (Bormuth et al., 2013). Interestingly, both transcription factors are abundantly expressed by postmitotic pyramidal neurons during embryonic development, albeit their expression levels decline in the postnatal life. *Neurod6* seems to be selectively expressed in a subset of pyramidal neurons, specifically those residing in the deeper layers of the adult mouse cortex; whereas *Neurod2* is expressed by all cortical pyramidal neurons irrespectively of their laminar position (Bormuth et al., 2013).

## Neurod Family in Cortical Development and Cortical Function

Pyramidal neurons (also known as cortical projection cells) are generated from progenitor cells located in the ventricular and subventricular zone of the dorsal telencephalon (see Figure 1; reviewed in Götz and Sommer, 2005; Elston and Fujita, 2014; Agirman et al., 2017). Soon after leaving the cell cycle, pyramidal neurons initiate a radial migration using the fibers of neighboring radial glial cells as a scaffold and cross an intermediate zone (IZ) on their way to reach their final destination within the developing cortex (reviewed in Rakic, 1995; Kriegstein and Noctor, 2004; He et al., 2015). After completing radial migration, pyramidal neurons settle in the cortical plate (CP) and undergo terminal differentiation (Gutierrez et al., 2004; Bianchi et al., 2013; Elston and Fujita, 2014). An interesting trait in cortical development is that the distinct projection neurons that form the mature cerebral cortex do not develop simultaneously but rather they are generated and migrate in a temporal order to populate the cortical plate in an inside-first and outside-last manner (reviewed in Angevine and Sidman, 1961; Rakic, 1974; Noctor et al., 2001; Buchsbaum and Cappello, 2019). This means that neurons residing in the deepest layers of the mature cerebral cortex are generated first during development and settle in the deepest part of the developing cortex. After pyramidal neurons form the deep layers of the cortex, superficially located projection neurons are generated and radially migrate to populate their corresponding upper layers (Rakic, 1974, 1995; Noctor et al., 2001; Kriegstein and Noctor, 2004; He et al., 2015). Once in the cortical plate, pyramidal neurons initiate dendritic arborizations and the projection of their axonal processes in a stereotyped manner, that is, they start with their axon outgrowth, fasciculation, pathfinding, and targeting of their appropriated neuronal partners (Figure 1).

bHLH genes cooperate to control transcriptional programs that select different aspects of neural progenitor cell biology and effectively determine neuron subtype identity. Proneural bHLH genes in the telencephalon, such as Neurog1 and Neurog2, activate transcriptional cascades of gene expression in progenitor cells that eventually lead to their terminal neuronal differentiation (Ge et al., 2006). Neurog1 and Neurog2 also contribute with the dorsalization of the telencephalon by suppressing the ventralization factor Ascl1 (Fode et al., 2000). The expression of Neurog 1 and Neurog2 is primarily restricted to the dorsal ventricular zone, although a few Neurog2 expressing cells can be observed outside this area (Ge et al., 2006). An important instructive function of Neurog2 is to promote a cortical neuron identity in differentiating cells of the dorsal telencephalon. Neurod4 is a known target of Neurog2 and dimerizes with it, forming Neurog2/Neurod4 heterodimers (Mattar et al., 2008). Neurog2/Neurod4 heterodimers accelerate the expression of particular transcriptional programs in the cortex that regulate neurogenesis. Mattar et al. (2008) have also shown that NeuroD4 and Neurog2 can independently act to regulate gene expression, albeit with a temporal delay. Similarly, Neurod4 can also form heterodimers with Neurog1 which are required for habenular neurogenesis. In the habenula, Neurod4 and Neurog1 depend on Pax6 expression downstream of Sonic hedgehog (Halluin et al., 2016).

Interestingly, the phosphorylation of Neurod4 limits its ability to drive neuronal differentiation during neurogenesis, which implies that post-transcriptional modifications finely tune the activity of bHLH transcription factors, such as Neurod4 (Hardwick and Philpott, 2015; Hardwick et al., 2019). In this context, a phospho-mutant Neurod4 increases its protein stability and enhances its chromatin binding when compared to wild-type Neurod4, which results in a transcriptional up-regulation of a wide range of target genes (Hardwick and Philpott, 2015). Lastly, Neurod4 has also been reported to be capable to reprogram human and mouse astrocytes, and when it is co-expressed with *Insm1* it is capable of driving glutamatergic neuron maturation (Masserdotti et al., 2015).

In the developing neocortex, Neurod1 has been shown to promote terminal neuronal differentiation in progenitor cells, although there exists a hierarchy in the sequential activation of transcription factors that regulate the transition from precursor cells to differentiated pyramidal neurons (Hevner et al., 2001, 2006; Muzio et al., 2002a, b; Hodge et al., 2008; Hodge and Hevner, 2011). Indeed, the sequential expression of the transcription factors *Pax6* → *Neurog2* → *Tbr2* → *Neurod1* → *Tbr1* correlates with the transition from primary progenitor cells into intermediate progenitors and ultimately into the generation of newborn glutamatergic pyramidal neurons. Outside the cerebral cortex, Neurod1 has also been shown to induce terminal neuronal differentiation. For instance, Boutin et al. (2010), using the olfactory periglomerular neuron lineage *in vitro*, showed that expression of *Neurod1* alone suffices to induce terminal differentiation in olfactory periglomerular progenitor cells (Boutin et al., 2010). *In vivo*, *Neurod1* overexpression in the periventricular region leads to the rapid appearance of postmitotic cells with morphological and molecular characteristics of mature neurons both in the subventricular zone and rostral migratory stream (Boutin et al., 2010). The function of Neurod1 in promoting terminal neuronal differentiation seems to be conserved in evolution and has been reported even in lower organisms like the worm *C. teleta* (Sur et al., 2017). Environmental enrichment also seems to induce *Neurod1* expression in the forebrain and to enhance neuronal activity. For example, studies in the juvenile Atlantic salmon (*Salmo salar*) showed that environmental enrichment upregulates *Neurod1* expression in their forebrain, which greatly improves their learning abilities (Salvanes et al., 2013). In mice, environmental enrichment leads to an increase in hippocampal volume and enhances dorsal-ventral differences in DNA methylation, including binding sites recognize by Neurod1, which seem to greatly promote adult neurogenesis (Zhang T.-Y. et al., 2018). In adult humans, *NEUROD1* expression increases in the cerebral cortex after a traumatic injury, which might indicate a protective mechanism play by Neurod1 in the postnatal cerebral cortex (Zheng et al., 2013).

Neurog1 and Neurog2 play an important function in suppressing *RhoA* expression just as cortical progenitor cells are about to leave the cell cycle, and this suppression is maintained in postmitotic neurons by the direct action of Neurod1 (Ge et al., 2006). The suppression of *RhoA* is critical for the migration of pyramidal neurons into the cortical plate (Ge et al., 2006). On the way to the cortical plate, pyramidal neurons also require Reelin, a protein secreted by the Cajal-Retzius cells that locate in the superficial marginal zone of the developing cortex. Neurod2 has been shown to control pyramidal neuron migration and Reelin signaling by direct regulation of *Cdk5r1*, *Lrp8* and the transcription factor *Cux1*, which in turn controls the differentiation of the upper layer (2/3 and 4) neurons (Bayam et al., 2015).

Neurod2 and Neurod6 are essential regulators of axonal navigation and axonal fasciculation in the mouse neocortex. For instance, the anterior commissure and the corpus callosum fiber tracts, which communicate the two cerebral hemispheres, are completely absent in *Neurod2* and *Neurod6* double mutant mice (Bormuth et al., 2013). Detailed inspections in *Neurod2* and *Neurod6* double mutant mice showed that callosal axons defasciculate in the subventricular zone during development and follow random trajectories into the ipsilateral cortex rather than growing toward the midline to contralaterally decussate (Bormuth et al., 2013). These axonal defects have been correlated with the dysregulation of the cell adhesion protein Cntn2 and the axonal receptor Robo1 (Bormuth et al., 2013). Furthermore, *Neurod2* mutant mice also exhibit deficits in their thalamocortical projections to different cortical areas, such as the somatosensory barrel cortex (Ince-Dunn et al., 2006). Furthermore, the ablation of *Neurod2* and *Neurod6* also results in reduced numbers of functional glutamatergic synapses and, consequently, in a diminished excitatory cortical network (Bormuth et al., 2013).

The development of dendrites and synapses is a fundamental process in the establishment of neuronal polarity and connectivity. In this regard, Neurod2 has been shown to regulate the structural and functional maturation of the hippocampal mossy fiber synapses *via* the regulation of the synaptic scaffolding protein PSD95 (Wilke et al., 2012). Neurod1 and Neurod2 can abrogate GABAergic differentiation directed by Ascl1, a well-known bHLH transcription factor critical for GABAergic neuron development. Furthermore, the forced expression of *Neurod2* in progenitor cells of the ventral telencephalon is sufficient to prevent their normal differentiation into GABAergic neurons (Roybon et al., 2010). In addition, Neurod2 regulates calcium signaling and homeostasis of mature neurons by controlling the expression of the *Stim1* gene that encodes for an ER calcium sensor (Guner et al., 2017). Abnormal dendritic spine remodeling and turnover from postnatal day 30, and onwards, was reported in *Neurod2* mutant mice, particularly in apical tuft dendrites of pyramidal layer 5 projection neurons of the somatosensory cortex (Runge et al., 2020a). Thus, Neurod2 is a nexus in the gene network that controls spine turnover in the postnatal cortex (Runge et al., 2020b).

Neural progenitor cells in the dorsal ventricular zone of the telencephalon also express *Neurod6*, some of which move into the subventricular zone and undergo multiple rounds of symmetrical and/or asymmetrical cell divisions to produce the set of neurons that reside in the upper cortical layers. Neurod6 positive progenitor cells in the subventricular zone are committed to generate glutamatergic neurons and might have evolved to expand the number of pyramidal neurons in the mammalian forebrain (Wu et al., 2005). Neurod6 has been shown to be central in the mitochondrial biogenesis during the early stages of neuronal differentiation. At these stages, Neurod6 appears to stimulate a maximal mitochondria mass accumulation which correlates with the onset of differentiation and lamellipodia formation in the axonal growth cone, as well as at the regions of axonal branching. This seems to be achieved by the transcriptional regulation of Neurod6 on genes encoding for cytoskeletal proteins, mitochondrial trafficking, regulators of membrane potential, and mitochondria chaperones (Uittenbogaard and Chiaramello, 2002, 2004, 2014; Kathleen Baxter et al., 2009; Uittenbogaard et al., 2010a, b; Baxter et al., 2012).

Neurod6 might also confer cellular tolerance to mitochondrial stressors and oxidative stress, which is critical to prevent neurodevelopmental disorders and neurodegenerative diseases, such as the autism spectrum disorder or Parkinson’s disease. The long–term consequences of early life stress on adult pathological states are associated with significant changes in DNA methylation and deregulation of miRNAs. *miR-30a-5p* regulates hundreds of downstream targets, including *Neurod6*, which may represent an important biological signature associated with the risk to develop psychiatric disorders as a consequence of exposure to early life adversities (Cattaneo et al., 2020). Neurod1 has also been shown to be critical for neuronal plasticity and increased levels of *Neurod1* expression are triggered in the murine hippocampus after chronic or mild stress (Boulle et al., 2014). During neuronal differentiation, DNA demethylation-reprograming events are also associated with Neurod2 genome-wide binding (Hahn et al., 2019). In particular, it has been shown that highly methylated genomic regions in neuronal progenitor cells become demethylated after the onset of *Neurod2* expression, and this coincides with the transition from proliferative progenitor state to differentiated neurons (Hahn et al., 2019). Furthermore, it has also been recently reported that maternal hyperglycemia increases H3K14 acetylation levels at Neurog1 and Neurod2 binding sites. Enhanced and premature expression of Neurog1 and Neurod2 eventually leads to an earlier differentiation of progenitor cells, which accelerates the genesis of newborn neurons in the cerebral cortex (Ji et al., 2019). Therefore, Neurod factors appear to display a pioneer function in remodeling chromatin. In keeping with this, Pataskar et al. (2016) recently demonstrated the pioneer function of Neurod1 in chromatin remodeling (Pataskar et al., 2016). The pioneer function of Neurod1 seems to be responsible for the potentiation of mineralocorticoid receptor-mediated transcription in the hippocampus, which has been suggested to act as a neuronal protective mechanism against the development of psychopathologies and, in particular, mood disorders (van Weert et al., 2019). In addition, Neurod1 has also been shown to reprogram striatal non-reactive astrocytes into neurons, albeit the reprogramming function of Neurod1 seems to be less efficient in cortical non-reactive astrocytes (Agirman et al., 2017; Liu et al., 2020).

Over two decades ago, Schwab et al. demonstrated that Neurod1 and Neurod6 are required for terminal neuronal differentiation in the hippocampus (Schwab et al., 1998, 2000). In *Neurod1* and *Neurod6* double mutant mice, the granule cells that are destined to populate the hippocampal dentate gyrus can be generated, but they fail to properly mature and display several phenotypes that include the lack of normal sodium currents, small dendritic arborization, and alterations of the entorhinal and commissural axonal projections (Schwab et al., 2000). Neurod1 has also been reported to play key functions in the survival and differentiation of newborn neurons in the subgranular and subventricular zones of the adult hippocampus (Gao et al., 2009). In keeping with this data, Roybon and others have also reported a key regulatory role for the Neurog2 and Neurod1 heterodimer complexes in controlling neuronal commitment and hippocampal neuroblast formation both in embryonic and in postnatal neurogenesis (Roybon et al., 2009). Specifically, Neurog2 and Neurod1 heterodimers control progenitor cell production and the amplification of granule neuron progenitors, but they are not required for the acquisition of their granule cell identity (Roybon et al., 2009). In the hippocampus, Neurod1 seems to induce the cell cycle exit of progenitor cells and to promote a rapid neuronal maturation of their progeny, maturation that seems to be reinforced by the expression of Neurod2 in differentiated hippocampal neurons (Roybon et al., 2009).

## Neurod Family in The Development of The Posterior Neural Tube

The Neurod family also plays critical functions in the development of the posterior nervous system, that is the cerebellum, brainstem, and the spinal cord. After the onset of neural induction, the brainstem and spinal cord adopt their posterior identity by responding to instructive patterning signals generated from specialized cell centers located within the neural tube as wells as in surrounding tissues. These patterning centers produce fibroblast growth factors, bone morphogenetic proteins, retinoids, and Wnt proteins, which are capable to diffuse over long distances to carry out their instructive function (Doniach, 1995; Lumsden and Krumlauf, 1996; Stern, 2005). The most salient outcome of this early patterning is the generation of distinct anterior-posterior segments characterized by and overlapping as well as differential expression of transcription factors, predominantly members of the Hox family (Philippidou and Dasen, 2013). In the brainstem, for example, seven units called rhombomeres (rhombomere 1–7) develop, whereas four units (cervical, thoracic, lumbar, and sacral) are specified in the spinal cord of humans and mice (Trainor and Krumlauf, 2000). Each rhombomeric and spinal cord unit is further patterned along their dorsoventral axis by the action of diffusible morphogens emanating from a dorsal and a ventral group of specialized cells that act as organizers, the roof, and floor plate. These two organizers antagonistically act and exert their function via the secretion of Sonic hedgehog (by the floor plate) and bone morphogenic proteins and Wnt proteins (by the roof plate; Roelink et al., 1995; Liem et al., 1997; Ulloa and Marti, 2010). These signals diffuse from the roof and floor plate forming concentration gradients that differentially act upon progenitor cells located at different distances from the signal source and along the dorsal-ventral axis of the neural tube. It is thus the spatial position of progenitor cells within the neural tube that determines their response to morphogenic signals. Progenitor cells then respond to these signals, in a dose-dependent manner, and differentially express particular sets of transcription factors, among these the Neurod family and several other bHLH factors.

This is the case of the cerebellum that develops from the dorsal part of rhombomere 1 (known as the cerebellar anlage), which directly receives instructive signals from the roof plate (Millet et al., 1996; Broccoli et al., 1999; Chizhikov et al., 2006; Butts et al., 2014a, b). The cerebellar anlage contains two distinct germinal zones, the ventricular zone and the rhombic lip, which produce all GABAergic and glutamatergic cerebellar neurons, respectively (Hallonet et al., 1990; Alder et al., 1996; Wingate and Hatten, 1999; Hoshino et al., 2005; Millen et al., 2014; Yamada et al., 2014). Rhombic lip progenitor cells generate three distinct neuronal populations in a stereotyped temporal order. The first generation of glutamatergic neurons occurs between embryonic days 10.5 and 13.5 (in mice), and during this period deep cerebellar neurons are generated. A subsequent generation of glutamatergic neurons occurs between embryonic day 13.5 and birth, throughout this time external granular cell layer cells become specified, these cells are the precursors of the granule cells that develop in the early postnatal life. Lastly, unipolar brush cells become specified from the rhombic lip between embryonic day 15.5 and the first day of postnatal life (Ben-Arie et al., 1997; Gazit et al., 2004; Machold and Fishell, 2005; Englund et al., 2006; Fink et al., 2006; Machold et al., 2011; Yamada et al., 2014).

The generation of deep cerebellar neurons seems to largely depend on the action of the bHLH factor Olig3 (Lowenstein et al., 2021), whereas the production of external granular layer cells and unipolar brush cells is regulated by the bHLH factor Atoh1 (Ben-Arie et al., 1997; Gazit et al., 2004; Machold and Fishell, 2005; Wang et al., 2005). Neurod1 has long been known to play a critical role in the differentiation of granule cells, mainly in postnatal life (Gao et al., 2009). Deletion of Neurod1 greatly disrupts differentiation of these cells by prolonging the proliferation of their external granular layer cell progenitors and, in parallel, by inducing apoptosis in the developing cerebellum (Miyata et al., 1999; Pan et al., 2009). The extended proliferation and lethality of Neurod1-deficient external granular layer cell progenitors might result from the loss of the pioneer and proneural function that Neurod1 exerts in these progenitor cells by mediating, among other molecular cascades, the expression of different elements of the Notch signaling pathway (Pataskar et al., 2016). Indeed, expression of Neurod1 is known to drive terminal neuronal differentiation in external granular layer cell progenitor cells both in development and in the postnatal life of mammals and other vertebrates, such as in *Xenopus* (Cho and Tsai, 2004; Boutin et al., 2010; D’Amico et al., 2013; Butts et al., 2014a; Hanzel et al., 2019). Furthermore, a recent report shows that elevated levels of *Neurod1* expression are sufficient to drive medulloblastoma cells into granule cell differentiation, which demonstrates that Neurod1 overrides oncogenic mutations present in medulloblastoma cells (Cheng et al., 2020). In spite of the substantial knowledge gained from the study of Neurod1 function in cerebellar development, less is known about the function of other members of the Neurod family in cerebellar development and cerebellar function. However, an early study using *Neurod2* mutant mice showed that these mutants correctly develop until about the second week of postnatal life, after which they began exhibiting ataxia and the failure to thrive, which seems to be indicative of a cerebellar dysfunction (Olson et al., 2001). More recently, Pieper and colleagues (2019) reported on a critical function of Neurod2 in promoting survival of both granule cells and inhibitory neurons (particularly basket and stellate cells) that originate from the ventricular zone (Pieper et al., 2019). The analysis of *Neurod2* mutant mice seems to indicate that Neurod2 might have an important function in cerebellar inhibitory neuron function, as well as in the axonogenesis and synaptic formation of inhibitory cerebellar neurons onto Purkinje cells (Pieper et al., 2019).

Work over the last two decades has also shown the great influence of the Neurod family in other regions of the posterior nervous system, which include the midbrain, the hindbrain, and the spinal cord. Progenitor cells in the ventral midbrain express high levels of *Neurod1*, and the combinatorial expression of *Neurod1* with other bHLH factors sub-specifies different neuronal populations emanating from this area, some of which retain *Neurod1* expression (Park et al., 2006; Arimura et al., 2019; Ásgrímsdóttir and Arenas, 2020; Poulin et al., 2020). Furthermore, Neurod1 and Neurod6 have been recently reported to have a critical function in the development of particular dopaminergic midbrain neurons (Khan et al., 2017). Specifically, Khan and colleagues analyzed *Neurod6* and *Neurod1* double mutant mice, and found that these genes are required for the survival of dopaminergic midbrain neurons located in the ventral tegmental area, particularly those that project to the intermediate and dorsal regions of the septum (Khan et al., 2017).

Unlike the neurogenic function that the Neurod family has in the developing cortex, cerebellum, and the midbrain; in the hindbrain and spinal cord Neurod1, Neurod2 and Neurod6 seem to mainly regulate the correct specification of discrete subpopulations of inhibitory interneurons (Hernandez-Miranda et al., 2017). In the hindbrain and spinal cord, Ptf1a-expressing progenitor cells generate all inhibitory neurons, which are known to co-express the homeodomain proteins Lbx1, Pax2, and Lhx1/5. Interestingly, these inhibitory interneurons do not uniformly maintain the expression of these transcription factors during their maturation and greatly vary in their expression, indicating that differential expression of such factors might reflect distinct subpopulations of inhibitory neurons (Pillai et al., 2007). Indeed, available evidence illustrates that Neurod1, Neurod2, and Neurod6 secure the specification of dynorphin+ and galanin+ inhibitory interneurons, whereas Lhx1/5 instruct a NPY+ inhibitory fate (Bröhl et al., 2008).

## Neurod Genes in Human Neurological Disorders

The first report of human patients with a mutation on a gene of the Neurod family came in 2010 by Rubio-Cabezas and others (Rubio-Cabezas et al., 2010). In this study, the authors reported on two distinct homozygous mutations in *NEUROD1* that were found in two unrelated probands diagnosed with permanent neonatal diabetes and neurological abnormalities. The identified mutations correspond to frameshift mutations that predictably generate truncated proteins, without affecting their DNA-binding domain. Neurologically, both patients presented with learning difficulties, cerebellar hypoplasia, profound sensorineural deafness, and visual impairment due to severe myopia and retinal dystrophy. Thus, the deficits observed in *NEUROD1*-deficient patients resemble those seen in *Neurod1* mutant mice, which include pronounced cortical, cerebellar, brainstem, and spinal cord impairments (see above). More recently, Sega and colleagues found de novo mutations in *NEUROD2* in two unrelated children diagnosed with early infantile epileptic encephalopathy and developmental delay (Sega et al., 2019). In keeping with this, an early onset of epilepsy has also been described in *Neurod2* deficient mice (Chen et al., 2016). In this context, Chen et al. suggested that Neurod2 tightly controls the inhibition/excitation balance of neuronal transmission in the mature cortex. Furthermore, deficiencies of Neurod2 function in the mouse brain cause a decrease in the cell-intrinsic excitability of excitatory pyramidal neurons (Chen et al., 2016). There are two transcriptional targets of Neurod2 that may contribute to these processes: gastrin-releasing peptide (GRP) and the small conductance, calcium-activated potassium channel, Sk2 (Kcnn2). The expression of both genes is greatly decreased in *Neurod2* mutant mice (Chen et al., 2016). Very recently, Runge et al. (2020b) described seven families with pathogenic *NEUROD2* mutations causing a variety of neurological disorders, such as autism spectrum disorders, intellectual disability, and speech delay. The authors of this study also suggest that behavioral deficits in social behavior, which are reminiscent of autistic disorders, can be found in *Neurod2* mutant mice. In addition, Spellman et al. identified a direct association of *NEUROD2* gene polymorphisms with changes in cognitive functions present in schizophrenic patients treated with antipsychotic drugs (Spellmann et al., 2017). It has also been shown that the lateral and basolateral amygdala nuclei fail to form in *Neurod2* mutant mice, and that these mice display deficits in emotional learning (Lin et al., 2005). In particular, Lin et al., found that Neurod2 is required for amygdala development and the regulation of the AMPA receptor, the γ subunit of the GABAA receptor, and the gene *Ulip1*, which are all involved in emotional learning (Lin et al., 2005).

The most salient phenotype arising from the loss of *NEUROD1* in humans is epilepsy. In mice, the ablation of *Neurod1* produces an epileptogenic phenotype associated with a malformation of the hippocampal dentate granule cell layer, which seems to result from an excessive cell death of the neurons forming this layer (Liu et al., 2020). Impaired neurogenesis and decreased expression of *NEUROD1/Neurod1* have also been demonstrated in the hippocampus of the Huntington’s disease R6/2 mouse model and in differentiated neural cultures derived from Huntington’s disease patients (Fedele et al., 2011). *NEUROD6* has been recently identified as a possible biomarker for the diagnosis of Alzheimer’s disease. Indeed, low expression levels of *NEUROD6/Neurod6* have been detected in postmortem Alzheimer’s patients and in Alzheimer’s mouse models using RNA sequencing datasets, microarray datasets, and meta-analysis (Hokama et al., 2014; Satoh et al., 2014; Li et al., 2015).

Despite the fact that *NEUROD1* expression levels have been reported not to be significantly changed in Alzheimer’s patients (Satoh et al., 2014), its overexpression into hippocampal progenitor cells increases dendritic spine density of hippocampal newborn neurons and results in a great improvement in spatial memory in the Alzheimer’s disease mouse model APPxPS1 (Richetin et al., 2015). Further studies by Richetin and others have also shown that Neurod1 promotes spinogenesis and mitochondrial availability at the vicinity of mature spines, and that this improves the integration and survival of adult-generated hippocampal neurons, which are severely impaired in the APPxPS1 mouse model (Richetin et al., 2017). These results provide a potential therapeutic approach to patients affected with Alzheimer’s disease. The half-life of Neurod1 can be increased by blocking its ubiquitin-dependent proteasomal degradation, which enhances the transcriptional programs mediated by Neurod1 during neuronal differentiation, but also those involved in neuronal maturation and synaptic transmission (de Wilde et al., 2016; Lee et al., 2020; Pomeshchik et al., 2020). Neurod1 has also been used to successfully reprogram reactive glial cells functional cortical neurons in stab-injured or Alzheimer’s disease mouse models and in adult non-human primates after ischemic stroke, which again offers the possibility to develop new therapeutical approaches for patients affected with Alzheimer’s disease (Guo et al., 2014; Ge et al., 2020).

## Conclusion

The bHLH superfamily of transcription factors is well conserved throughout evolution and plays critical roles in tissue development and tissue maintenance. Whereas many bHLH transcription factors display ubiquitous expression, a small fraction of them has a tissue-specific expression. The question of how different members of this superfamily were selected to carry out common and divergent cellular functions remains to be elucidated. In the developing nervous system, the subfamily of neural lineage bHLH transcription factors regulates a variety of biological functions that range from progenitor cell proliferation and survival to neuronal differentiation, neuronal migration, fate specification, axonal navigation, dendritic elongation, and synaptic formation. Some members of this subfamily (called proneural, including Ascl1, Neurog1, Neurog2, Neurod1, and Neurod4) have been shown to be able to remodel chromatin and induce neuronal differentiation in progenitor cells, but they are also capable of reprogramming differentiated non-neuronal cells into neurons (Castro et al., 2011; Wapinski et al., 2013, 2017; Chanda et al., 2014; Treutlein et al., 2016; Rao et al., 2021). While Ascl1, Neurog1, Neurog2, and Neurod4 are predominantly expressed in progenitor cells, other factors like Neurod1, Neurod2, and Neurod6 are expressed both in progenitors and retained in postmitotic neurons. This raises the question of whether the function of these factors differs in progenitor cells and in differentiated neurons. Future research may elucidate whether post-transcriptional regulations, such as phosphorylation, on Neurod1, Neurod2, and Neurod6 account for their functional restriction at different points in the life of a neuron. Recent work on Ascl1 (in neurons) or Myod1 (in muscle cells) has shown that these transcription factors have an oscillatory expression which accounts for the proliferation of progenitors cells, whereas the sustained expression of these factors drives cell differentiation (Imayoshi et al., 2013; Vasconcelos and Castro, 2014; Lahmann et al., 2019; Sueda et al., 2019; Zhang et al., 2021), whether this oscillatory behavior is common for all bHLH transcription factors is unknown. However, the Neurod family offers the possibility to deepen into the expression dynamics of bHLH factors as they are expressed both in progenitors and in their progeny. An early diagnosis of neurological diseases is central in their management. Recent discoveries suggest that the expression of distinct members of the NEUROD family could serve as biomarkers at the onset of various neurological diseases, such as Alzheimer’s disease, and also serve in the development of patient-oriented gene therapies.

## Author Contributions

ST, VT, and LH-M wrote the original draft and reviewed the literature. ST, VT, and LH-M revised the original draft. LH-M edited the final draft. All authors contributed to the article and approved the submitted final version.

## Conflict of Interest

The authors declare that the research was conducted in the absence of any commercial or financial relationships that could be construed as a potential conflict of interest.
